# Severe lingual tonsillar hypertrophy and the rationale supporting early use of wire-guided retrograde intubation

**DOI:** 10.4103/1658-354X.65120

**Published:** 2010

**Authors:** Kristopher Schroeder, Aimee Becker, Christopher Guite, George Arndt

**Affiliations:** *University of Wisconsin School of Medicine and Public Health, Department of Anesthesiology, Clinical Science Center, Madison, WI, USA*

**Keywords:** *Lingual tonsillar hypertrophy*, *retrograde intubation*, *difficult intubation*

## Abstract

An expanding body of literature exists which describes the airway challenges and management options for lingual tonsillar hypertrophy (LTH). The use of retrograde intubation to secure a patient‘s airway in the setting of LTH has been previously unreported and should be considered early in the event of a cannot intubate, cannot ventilate scenario. A 55-year-old man, who had previously been described as an easy intubation, presented an unexpected cannot intubate, cannot ventilate scenario secondary to LTH. Various noninvasive airway maneuvers were attempted to restore ventilation without success. We describe the advantages of early use of wire-guided retrograde intubation as an alternative to a surgical airway for obtaining a secure airway in a patient with LTH, in whom noninvasive airway management maneuvers have failed. Multiple different noninvasive approaches to management of LTH have been previously described including the laryngeal tube, laryngeal mask airway, and fiberoptic bronchoscopy. Unfortunately, none of these noninvasive airway maneuvers successfully ventilated this patient and an invasive airway became necessary. Retrograde intubation is a less invasive alternative to the surgical airway with potentially less risk for complications. Retrograde intubation may be particularly effective in the setting of LTH as it may stent open an otherwise occluded airway and allow passage of an endotracheal tube. Skillful use of this technique should be considered early as a viable option in any case of unexpected difficult intubation due to LTH.

## INTRODUCTION

Lingual tonsillar hypertrophy (LTH) is a life-threatening cause of unknown difficult intubations. It can rapidly progress to the cannot intubate, cannot ventilate scenario. We discuss the advantages of early application of wire-guided retrograde intubation as an alternative to a surgical airway in the cannot intubate, cannot ventilate scenario due to LTH when other modalities of airway management have failed.

## CASE REPORT

A 55-year-old, 86 kg, ASA 3, African American male presented at our institution for an elective robotic prostatectomy. The patient had a past medical history significant for Gleason grade IV cancer of his prostate, well-treated essential hypertension, and nonischemic dilated cardiomyopathy status-post orthotopic heart transplant 4 years ago. Preoperative evaluation revealed that at the time of his heart transplantation, that is 4 years back, the patient‘s airway management had been described as being easy to bag and mask ventilate and that he was easy to intubate with a Macintosh 3 blade.

Examination of the patient‘s airway revealed a wide mouth opening, a neck which extended well, Mallampati class I airway, thyromental distance of 6 cm, intact dentition, and a basal metabolic index (BMI) of 24.3. Following the establishment of intravenous access with an 18-gauge peripheral IV, the patient was brought to the operating suite where standard American Society of Anesthesiologists (ASA) monitors were applied. The patient was preoxygenated with a face mask for 5 minutes and anesthesia was subsequently induced with intravenous propofol, lidocaine, and fentanyl. Upon establishment of an easy bag-mask airway, the patient was given a 0.1 mg/kg dose of vecuronium in preparation for intubation. Initial attempts at direct laryngoscopy with a Macintosh 3 blade revealed abundant soft tissue protruding from the base of the tongue and vallecula, which entirely obstructed the view of the laryngeal anatomy. The obstructing tissue was immediately recognized as lingual tonsils and a single attempt at inserting an 8.0 cuffed endotracheal tube beneath the tissue was unsuccessful. Two subsequent attempts to directly insert an Eschmann stylet using Macintosh 3 and 4 blades were also unsuccessful. Insertion of both #5 and #4 classic laryngeal mask airways (LMAs) and #4 Proseal LMA failed to produce adequate ventilation. Following these airway maneuvers, a two-handed bag-mask technique was required to deliver adequate breaths. Two attempts at fiberoptic bronchoscopy through the facemask during mask ventilation revealed only friable soft tissue. The mask ventilation continued to become progressively more difficult and ultimately ineffective during fiberoptic bronchoscopy. An emergent call was placed to the on-call ENT surgeon after the decision was made to proceed with a surgical airway. Upon consultation with the ENT surgeon, it was decided to attempt retrograde intubation as although ventilation was impossible, oxygenation was maintained with an SpO_2_ over 93%.

A Cook retrograde intubation kit (Bloomington, IN, USA) was obtained and opened. The patient‘s neck was prepped with chlorhexidine. The trachea was localized through the cricothyroid membrane using an 18-gauge sheath-over-needle catheter and a saline-filled syringe. The catheter was advanced into the airway after air was aspirated from the trachea. The 0.038-inch diameter, Teflon-coated 110-cm J-type guide wire was directed cephalad through the catheter into the trachea and oropharynx. Magill forceps were used to grasp the guide wire out of the mouth. The guide wire was advanced through the skin to the indicator mark and locked in place at the skin with a hemostat. The 70-cm tapered retrograde exchange catheter was then advanced over the guide wire and into the airway. A 6.0-mm ID endotracheal tube was successfully passed over the exchange catheter and into the trachea. The guide wire was then removed by pulling it out through the skin. The retrograde catheter was advanced in the trachea and the 6.0-mm ID endotracheal tube advanced over it. The exchange catheter was then removed. Endotracheal tube position was confirmed by auscultation, capnography, and fiberoptic bronchoscopy. The 6.0-mm ID endotracheal tube was replaced by an 8.0-mm ID endotracheal tube using a Cook Adult Tube Exchanger (Bloomington, IN, USA). The remainder of the operation proceeded without incident and the patient was taken to the ICU intubated. He was extubated the following day without incident and recovered from the procedure with no adverse sequelae.

## DISCUSSION

LTH is being increasingly recognized as a cause of both unexpected difficult intubation and difficult mask ventilation. Lingual tonsils exist at the base of the tongue and are bounded by the epiglottis posteriorly, vallate papillae anteriorly, and tonsillar pillars bilaterally. LTH can be asymptomatic, but can be associated with vague symptoms including sore throat, dysphagia, snoring, globus sensation, and obstructive sleep apnea. The danger of LTH lies in the fact that traditional preoperative oropharyngeal examination fails to hint at the difficulties that may be encountered following induction of general anesthesia and muscle relaxation. In addition, following muscle relaxation lingual tonsils may act as a “ball valve” that prohibits antegrade ventilation or insertion of an endotracheal tube. Multiple approaches have been described in the literature to deal with difficult intubation and ventilation associated with LTH.

Matioc *et al*.[1] have described the use of a laryngeal tube in a patient with LTH. Laryngoscope insertion in their patient revealed LTH which blocked visualization of the epiglottis. Mask ventilation was adequate with an oral airway but insertion of a laryngeal mask airway was unable to effectively ventilate the patient. Insertion of a laryngeal tube produced effective ventilation and the patient was able to proceed with the surgical procedure without further incident. Postoperative otolaryngology examination revealed significant LTH which displaced the epiglottis posterior. Ojeda *et al*.[2] have described two cases of LTH where intubation with an LMA North America, Inc. San Diego, CA, USA failed and was only accomplished with the use of fiberoptic bronchoscopy.

Davies *et al*.[3] have reported three cases of LTH. In one case, a patient with a normal preoperative airway examination was unable to be successfully intubated with either direct laryngoscopy or fiberoptic bronchoscopy. Laryngeal mask airway initially provided sufficient ventilation and the authors chose to awaken the patient following reversal of muscle relaxation. Following removal of the LMA, the patient began to struggle with ventilation and rapidly desaturated. Mask ventilation with two hands and an oral airway at this point proved to be impossible; the anesthesiologists rapidly progressed with cricothyroidotomy. The patient recovered and postoperatively, enlarged lingual tonsils were confirmed as the etiology of the difficult intubation.

The most thorough examination of LTH was completed by Ovassapian *et al*.[4] They examined 33 patients who were found to be unexpected difficult intubations. Fiberoptic pharyngoscopy revealed LTH in all cases. Direct laryngoscopy could not accomplish endotracheal intubation in each case but every patient was successfully intubated via fiberoptic bronchoscopy. No patient experienced any long-term sequelae.

In the event of a cannot intubate, cannot ventilate situation, the 2003 ASA Practice Guidelines suggest early use of alternative airway management devices with the goal of minimizing stomach insufflation, limiting trauma to the upper airway, and providing ventilation and oxygenation. In our case, several methods of intubation were attempted following the ASA Practice Guidelines including direct laryngoscopy with and without bougie, several sizes of LMAs, and fiberoptic bronchoscopy early on in the course of attempted airway management. Both disposable and classic LMAs failed to provide effective ventilation. As mask ventilation proved progressively more difficult, invasive airway management became necessary and once we could no longer ventilate or intubate the patient, it became necessary to intubate the trachea via retrograde intubation. Had we not decided to secure the patient‘s airway early on via the wire-guided retrograde method, patient decompensation and the requirement for a surgical airway would have surely occurred. We report this case as it has not been reported in the past and it demonstrates that delaying the application of the retrograde method after failure of traditional antegrade methods could have disastrous consequences.

Retrograde intubation has advantages as an invasive airway management technique as it is potentially less dangerous and less invasive than cricothyrotomy and tracheostomy. An early description of retrograde intubation was provided by Butler and Cirrillo in 1960.[5] They describe successful passage of a No. 16 French catheter cephalad through a preexisting tracheotomy. An endotracheal tube was then advanced over the catheter.

In the setting of LTH, the airway can be occluded by either the enlarged tonsils or an epiglottis that is displaced in a posterior direction. These two factors could combine to create a situation where an antegrade intubation becomes difficult or impossible. Retrograde intubation may effectively stent open the airway and allow passage of an endotracheal tube. Complications of retrograde intubation can include bleeding, subcutaneous emphysema, pneumomediastinum, difficulty passing the endotracheal tube past vocal cords, sore throat, a prolonged time required for intubation, and potentially a low success rate. Weksler *et al*.[6] have reviewed the cases of 24 patients who underwent retrograde intubation. They found that retrograde intubation was easily accomplished in all patients with minimal side effects. This is in contrast to a review done by Gill *et al*.[7] which demonstrated that retrograde intubation was only successful in four out of eight patients and two attempts took longer than 5 minutes.

In summary, we present percutaneous wire-guided retrograde intubation as a viable option in the cannot intubate, cannot ventilate scenario secondary to LTH. We also advocate that early application of wire-guided retrograde intubation should be considered in the cannot intubate, cannot ventilate scenario secondary to LTH.
